# Gap Junctional Communication in Osteocytes Is Amplified by Low Intensity Vibrations In Vitro

**DOI:** 10.1371/journal.pone.0090840

**Published:** 2014-03-10

**Authors:** Gunes Uzer, Suphannee Pongkitwitoon, Cheng Ian, William R. Thompson, Janet Rubin, Meilin E. Chan, Stefan Judex

**Affiliations:** 1 Department of Biomedical Engineering, Stony Brook University, Stony Brook, New York, United States of America; 2 Department of Medicine, University of North Carolina, Chapel Hill, North Carolina, United States of America; Ohio State University, United States of America

## Abstract

The physical mechanism by which cells sense high-frequency mechanical signals of small magnitude is unknown. During exposure to vibrations, cell populations within a bone are subjected not only to acceleratory motions but also to fluid shear as a result of fluid-cell interactions. We explored displacements of the cell nucleus during exposure to vibrations with a finite element (FE) model and tested in vitro whether vibrations can affect osteocyte communication independent of fluid shear. Osteocyte like MLO-Y4 cells were subjected to vibrations at acceleration magnitudes of 0.15 g and 1 g and frequencies of 30 Hz and 100 Hz. Gap junctional intracellular communication (GJIC) in response to these four individual vibration regimes was investigated. The FE model demonstrated that vibration induced dynamic accelerations caused larger relative nuclear displacement than fluid shear. Across the four regimes, vibrations significantly increased GJIC between osteocytes by 25%. Enhanced GJIC was independent of vibration induced fluid shear; there were no differences in GJIC between the four different vibration regimes even though differences in fluid shear generated by the four regimes varied 23-fold. Vibration induced increases in GJIC were not associated with altered connexin 43 (Cx43) mRNA or protein levels, but were dependent on Akt activation. Combined, the in silico and in vitro experiments suggest that externally applied vibrations caused nuclear motions and that large differences in fluid shear did not influence nuclear motion (<1%) or GJIC, perhaps indicating that vibration induced nuclear motions may directly increase GJIC. Whether the increase in GJIC is instrumental in modulating anabolic and anti-catabolic processes associated with the application of vibrations remains to be determined.

## Introduction

Gap junctions formed by connexins play an important role in cell signaling and tissue function by enabling the passing of ions and intracellular signaling molecules via transmembrane channels in various organ systems [1]–[3]. In bone, connexin 43 (Cx43) is the most common connexin, present in osteoblasts, osteoclasts, stromal cells and osteocytes [4]–[8]. Connexin 43 can serve as an open ended hemi-channel to secrete signaling molecules such as NO, PGE_2_ and Ca^2+^ [9]–[13] or provide functional communication between resident bone cells via gap junctions, a process that is critical for coordinating bone remodeling and cell function [14]–[19].

Gap junctional intercellular communication (GJIC) is also important for cell mechanotransduction. Both fluid shear stress and mechanical strain increase GJIC between bone cells [20]–[23]. Osteocytes, embedded within the bone matrix, are well positioned to effectively use GJIC to communicate mechanically derived responses. Consistent with the hypothesis of osteocytes being the sensory cells that orchestrate the response of osteoblastic and osteoclastic effector cells [24]–[26], mechanical perturbation of osteocytes can regulate osteoblast function through gap junctions [27]. Thus, GJIC may play an important role in relaying mechanically derived signals to other cells such as osteoblasts [28] or vice versa.

Mechanical signals including fluid flow and mechanical stretch have been shown to regulate Cx43 function and GJIC [29]–[32]. In osteocytes, Cx43 activity is regulated by fluid flow through PI3K/Akt signaling [33], inhibiting glycogen synthase kinase-3β (GSK-3β) a critical component of the β-catenin degradation complex. Under fluid flow, PI3K mediated Akt activation is controlled via integrins [34] and focal adhesion kinase (FAK) [35] while mechanical stretch activates Akt through a PI3K independent mechanism [36], [37]. Low intensity vibrations, a mechanical signal anabolic and/or anti-catabolic to bone [38]–[42], can also increase β-catenin levels through inhibition of GSK-3β [42], suggesting that perhaps vibrations regulate Akt activation and thereby modulate GJIC.

Vibrations applied either in vivo or vitro create a complex cellular mechanical environment that is dependent on vibration magnitude (acceleration) and frequency. In vivo, vibrations can cause significant fluid shear on trabecular bone surfaces [43], [44] in the absence of significant matrix strain levels [38], [45]. We previously showed that vibration induced fluid shear stresses in vitro can be finely tuned by vibration frequency and acceleration [46]. In addition to fluid shear, dynamic accelerations ostensibly cause out-of-phase motions of the nucleus [38], another potential signal transduction mechanism by which vibrations may produce biochemical signals. In support of the hypothesis that vibrations can be sensed through nuclear motions, PGE_2_ and NO responses of osteoblast-like cells are acceleration rate dependent [47]. Also, vibration induced transcriptional activity of cytoskeletal regulators, including Arp2/3 complex and RhoA, was correlated with acceleration magnitude rather than fluid shear [48].

Here we investigated if vibrations increase GJIC and whether these changes are related to a specific physical variable that defines the nature of the oscillatory signal. Specifically, we hypothesized that vibration induced accelerations generate larger relative nuclear motions than vibration induced fluid shear and that the mechanically modulated increase in GJIC is independent of fluid shear through an Akt dependent pathway.

## Methods

### Experimental design

We addressed the question whether vibrations affect osteocyte communication independent of fluid shear. Using previously established methods to quantify vibration induced fluid shear stress [46], we applied four different vibration regimens *in vitro*, each exposing adherent cells to distinct levels of fluid shear stress.

In silico, a finite element (FE) model of an adherent cell was constructed to identify maximal displacements of the cell nucleus caused by vibration induced accelerations or vibration induced fluid shear. In vitro, calcein stained MC3T3 (ATCC, CRL-2593, VA) cells were parachuted onto osteocyte like MLO-Y4 [49] cells via a dye transfer assay [31]. MC3T3 cells were used as donor cells due to their ability to create functional gap junctions with MLO-Y4 cells within 15 minutes [28]. Following a given vibration regimen, the percentage of total GJIC positive MLO-Y4 cells (GJIC+) was compared to non-vibrated controls using flow cytometry. Cell-to-cell communication through gap junctions was verified with 18α-glycyrrhetinic acid (18α-GA), a gap junction inhibitor. To test whether Akt activation is involved in altered GJIC, osteocytes were pre-treated with Akt inhibitor Akti1/2 and both GJIC+ (flow cytometry) and Akt activation (Ser473, western blots) were measured following exposure to vibrations.

### Application of high-frequency oscillations and determination of fluid shear

The horizontal vibration system generating the mechanical signals is described in detail elsewhere [46]. Briefly, an actuator was attached to a linear frictionless slide. This system can simultaneously vibrate up to three cell culture plates. Vibrations were applied at peak magnitudes of 0.15 g or 1 g and frequencies of either 30 Hz or 100 Hz, resulting in four distinct oscillatory regimes. Cells were oscillated for 30 min at RT. Control samples were handled exactly the same except that the actuator was not turned on. During vibrations, out-of-phase motions of the cell culture medium within the well and the resulting fluid shear stress were determined with an experimentally validated finite element model [46]. At 100 Hz and 0.15 g, peak fluid shear stresses reached 0.04Pa, a level that increased to 0.14Pa at 30 Hz/0.15 g, 0.28Pa at 100 Hz/1g, and 0.94Pa at 30 Hz/1 g.

### Finite element modeling of a cell

An adherent cell was modeled with FE software (Abaqus 6.9.1, Simula, RI) and vibration induced nuclear displacements were estimated via dynamic stress analysis. Cell geometry was adopted from previous models of adherent cells [50], [51] with a cell contact radius of 19.2 µm and a cell height of 7.6 µm. The nucleus was modeled as an ellipsoid with a major axis of 7.5 µm and a minor axis of 2.5 µm. These cell dimensions are comparable to those from confocal images of osteocytes within the lacunar-canalicular network [52].

**Figure 1:**
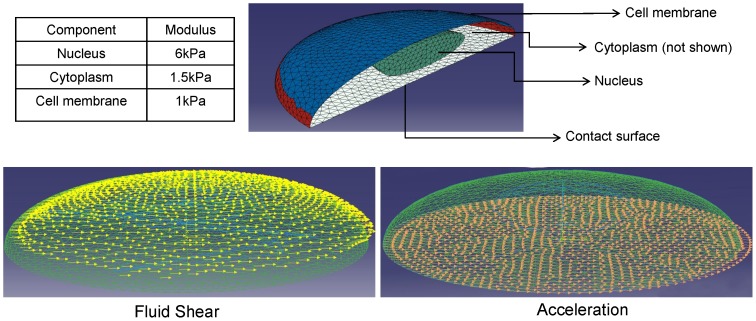
Finite element model of an adherent cell to identify vibration induced nuclear displacements. The elastic FE model comprised the cell membrane, cytoplasm and nucleus (top). Vibration induced fluid shear and accelerations were evaluated in separate dynamic simulations. Fluid shear was simulated by applying dynamically oscillating forces to the cell membrane (bottom left). Oscillatory accelerations were applied directly to the cell contact surface (bottom right).

The modeled cell comprised three components: cell membrane, cytoplasm, and nucleus (**Fig. 1**). Material properties were assumed to be elastic because mechanical vibrations were applied at a frequency of 30 Hz or higher, well below the measured viscoelastic relaxation times of about 40 s [53]. Density ratios (1∶1.2∶0.4) were approximated from refractive index measurements between the cytoplasm, nucleus, and cell membrane (triglycerides) [54], [55]. The density of the cytoplasm was assumed to be 50% greater than that of water (1500 kg/m^3^). A bending modulus 1.17×10^−19^Nm was assigned to the cell membrane [56]. Based on atomic force microscopy (AFM) measurements of an osteoblast nucleus, nuclear stiffness was set at 6kPa [53]. Since the nucleus was found to be four times stiffer than the cytoplasm [57], cytoplasm stiffness was set at 1.5kPa. All simulations were repeated for 50% and 300% of the initially assumed material properties, covering a nuclear modulus range of 3–18kPa, similar to the previously reported range of 2.6–8.3kPa across osteoblasts and osteocytes [58].

To calculate cellular deformations produced either by vibration induced accelerations or by vibration induced fluid shear, two distinct simulations were performed (**Fig. 1**). For acceleration simulations, the cell substrate was subjected to sinusoidal motions in a horizontal plane with accelerations of 0.15g or 1g and frequencies of 30 Hz or 100 Hz. For fluid shear simulations, the cell substrate was fixed so that it was not able to move. Sinusoidal dynamic forces with magnitudes that matched the vibration induced fluid shear magnitudes [46] were applied to the cell membrane.

Total force applied to the cell membrane was estimated from previously quantified peak fluid shear stresses [50]. At 0.94Pa fluid shear, for example, the total tangential force acting on the cell surface was 0.94pN/µm^2^×1470 µm^2^  = 1381.8pN, where 1470 µm^2^ is the total surface area of the cell. Total force was equally divided between all 5768 elements of the cell membrane. Nuclear motion (relative to input signal) was selected as an outcome variable and defined as the relative motion between the nucleus center and the cell contact surface in the direction of the vibration (horizontal).

### Cell culture

MLO-Y4 cells [59] were graciously donated by Dr. Lynda F. Bonewald. Cells were cultured in 75 cm^2^ cell culture flasks (BD Biosciences, NJ) at a density of 5000cell/cm^2^. α-MEM (Invitrogen, NY) supplemented with 2.5% fetal bovine serum (FBS, Gibco, CA), 2.5% bovine calf serum (BCS, Thermo Scientific, IL) and 1% Penicillin-Streptomycin (PS, Gibco, CA) was used as cell culture medium. MC3T3 cells were plated at 5000cell/cm^2^ in 100 mm cell culture dishes (Corning Inc., NY) and maintained in α-MEM supplemented with 10% FBS and 1% PS. All cells were maintained at 37°C and 5% CO2 and passaged at 70% confluency.

### Blocking reagents

Cells were either pre-treated with 75 µM 18α-GA gap junction blocker for 3 h (Sigma, MO) or with 40 µM Akt inhibitor AKTi1/2 for 1 h (Sigma, MO). Controls were treated with DMSO only. Blockers were maintained in cell culture medium during experiments. To confirm that a dose of 75 µM 18α-GA was not toxic, we performed a toxicity analysis with MLO-Y4 cells. Following pretreatment, cells were exposed to 75 µM 18α-GA (n = 6 per group). Using a live/dead cell cytotoxicity kit (Invitrogen, L-3224), cells were stained with calcein (4µM) and EtBr (2 µM). Immediately after staining, cells were washed with PBS, trypsinized and sorted using a flow cytometer (10,000 cells per sample).

### Parachute assay

MLO-Y4 cells were seeded in 24-well plates (CLS3527, Corning Inc.) coated with 0.15 mg/ml rat tail collagen I (Cell Applications Inc., CA) using 0.5 ml of culture medium at a density of 10,000 cell/cm^2^. Cells were incubated for 72 h to reach 80–90% confluence. Four hours prior to vibration treatment, MC3T3 cells (70% confluent) were treated with 1 μM calcein AM for 30 min according to the manufacturer's instructions (L-3224, Invitrogen) and returned to the incubator. Immediately after vibration treatment, donor MC3T3 cells were parachuted on top of MLO-Y4 at a ratio of 1∶500. Plates were returned to the incubator for 1 hr to allow GJIC to occur. Cells were then processed for flow cytometry to measure calcein positivity, for RNA extraction to measure transcriptional levels, or for western blotting to measure changes in protein levels. Experiments were repeated at least three times with a sample size of six per group. Results from individual experiments were pooled yielding a minimum of n = 18 per group.

### Flow cytometry

A total of 5000 live cells were analyzed by flow cytometry (FACScan, BD) capable of reading calcein 495/515 nm spectra. Cells that were between negative controls (no calcein) and positive controls (only donor cells) on the fluorescence intensity scale were selected as GJIC positive cells (GJIC+). The effect of vibration treatment was quantified through the relative difference of total GJIC+ cells between the treated and the control group. Flow cytometry analysis was performed using Flowjo software (Tree Star Inc., OR). Calcein dye transfer between cells was also visualized by fluorescence microscopy (Zeiss, NY).

### RNA extraction and qPCR

Cells were lysed with 600 ml of TRIzol (Ambion, TX) and stored in −80°C. Total RNA was isolated (RNeasy Mini Kit, Qiagen, CA) and its quality and concentration were determined (NanodropND-1000, Thermo Scientific, NY). Upon reverse transcription (High Capacity RNA to cDNA kit, Applied Biosystems, CA), RT-PCR was performed (Step-One Plus, Applied Biosystems, CA) using Taqman primer probes (Applied Biosystems, CA) for Cx43 (GJA-1) and GAPDH which served as referent. Expression levels were quantified with the delta-delta CT method [60] and results were reported relative to non-vibrated control.

### Western Blotting

Whole cell lysates were prepared using an radio immunoprecipitation assay (RIPA) lysis buffer (150 mM NaCl, 50 mM Tris HCl, 1 mM EDTA, 0.24% sodium deoxycholate,1% Igepal, pH 7.5) to protect samples from protein degradation. NaF (25 mM) and Na3VO4 (2 mM), Aprotinin, leupeptin, pepstatin, and phenylmethylsulfonylfluoride (PMSF) were added to the lysis buffer. Whole cell lysates (20 μg) were separated on 9% polyacrylamide gels and transferred to polyvinylidene difluoride (PVDF) membranes. Membranes were blocked with milk (5%, w/v) diluted in Tris-buffered saline containing Tween20 (TBS-T, 0.05%). Blots were then incubated overnight at 4°C with appropriate primary antibodies. Antibodies included those targeting Akt (#4685), pAkt (Ser-473, Cell Signaling, Danvers, MA), Cx43 (abcam, gja1,ab11370, MA) and tubulin (abcam, ab7291, MA). Following primary antibody incubation, blots were washed and incubated with horseradish peroxidase-conjugated secondary antibody diluted at 1∶5,000 (Cell Signaling) at RT for 1 h. Chemiluminescence was detected with ECL plus (Amersham Biosciences, Piscataway, NJ) and densitometry was performed via NIH ImageJ software.

### Statistical analysis

Results were presented as mean ± SEM. Differences between groups were identified by one-way analysis of variance (ANOVA) followed by Newman-Keuls post-hoc tests (flow cytometry and westerns). Non parametric Spearman Rank tests were used to assess the association between GJIC and mechanical variables obtained from the FE model. P-values of less than 0.05 were considered significant.

## Results

### Nuclear motions determined by finite element modeling

To explore cellular deformations during vibration, we generated a FE model of an elastic cell. Considering the greater stiffness and density of the nucleus, nuclear displacement was selected as outcome variable and measured under the application of either dynamic acceleration or fluid shear (**Table 1**). Nuclear displacements were found to be modulated by the magnitude of the applied acceleration. When averaged across 30 Hz and 100 Hz vibration frequencies, nucleus displacement was 127 nm at 0.15 g and 780 nm at 1 g. The difference between 30 Hz-0.15 g and 30 Hz-1 g was 27% greater than the difference between 100 Hz-0.15 g and 100 Hz-1 g groups, demonstrating that, at least to some degree, vibration frequency interacts with acceleration to determine nuclear displacement.

**Table 1:** Nuclear displacements induced by either vibration induced accelerations or vibration induced fluid shear.

Frequency	Cytoplasm stiffness	Acceleration magnitude		Fluid shear stress			
		*0.15g*	*1g*	*0.04Pa*	*0.14Pa*	*0.28Pa*	*0.94Pa*
100Hz	0.75kPa	352nm	1840nm	0.096nm	-	6.72nm	-
	1.5kPa	137nm	717nm	0.044nm	-	3.08nm	-
	4.5kPa	45nm	283nm	0.013nm	-	0.92nm	-
30Hz	0.75kPa	258nm	1554nm	-	3.36nm	-	2.50nm
	1.5kPa	117nm	844nm	-	1.54nm	-	10.34nm
	4.5kPa	37nm	226nm	-	0.46nm	-	3.10nm

Nuclear displacement was inversely proportional to cell stiffness. Averaged across all groups, decreasing cell stiffness by 50% increased nuclear displacement by 229±17% while increasing stiffness by 300% decreased nuclear displacement by 67±3%. Relative differences between individual groups were also stiffness dependent. At a nuclear stiffness of 6kPa, the 30 Hz-1 g treatment group had a 17% greater nuclear displacement than the 100 Hz-1 g group. When cell stiffness was decreased by 50% or increased by 300%, nuclear displacement in the 100 Hz-1 g was 16% and 21% larger than the corresponding displacement in cells exposed to 30 Hz-1 g. Accelerations caused 10 to 100 times larger nuclear displacements compared to fluid shear induced by the same vibration frequency/acceleration (**Table 1**).

### Gap junctional communication

**Figure 2:**
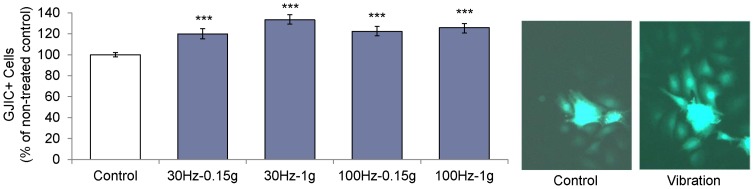
Vibrations increase gap junctional communication (GJIC) in MLO-Y4 cells. MLO-Y4 cells were exposed to one of four distinct vibration regimes and GJIC+ cell number was compared to non-vibrated controls (left). Averaged across the four vibrational signals, GJIC+ cell number was 25% greater than in controls (p<0.001) without significant differences between vibrated groups. Qualitative fluorescent microscopy revealed that vibrated cells communicated farther than controls (right). ***: p<0.001 against control.

**Figure 3:**
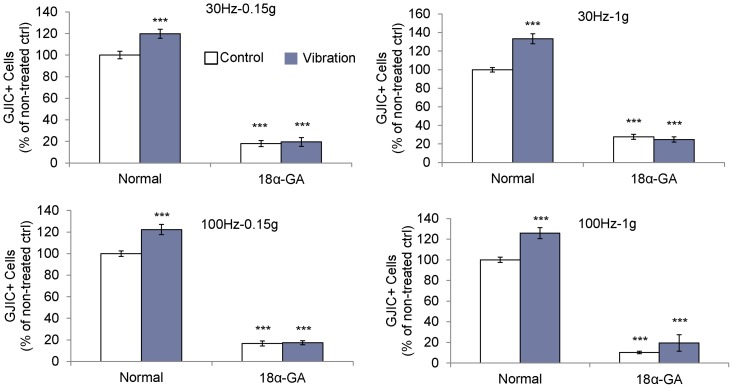
Vibration induced calcein transference is gap junction specific. When gap junction function was blocked with 75 µM of 18α-GA, GJIC+ cell number was significantly reduced compared to non-blocked groups (Normal) and none of the four vibration frequency/acceleration combinations increased the number of GJIC+ cells. ***: p<0.001 against control.

Calcein positive cells (excluding donor cells) were measured and compared to controls after exposure to one of four vibration regimes for 30 min followed by 1 h incubation. All vibration regimes significantly increased (p<0.001) the number of GJIC+ cells compared to non-vibrated controls **(** **Fig. 2**). Cells vibrated at 30 Hz-1g showed the greatest increase in calcein transference (33±5%, p<0.001) but no significant differences were observed between individual vibrated groups. Microscope images qualitatively showed that vibrations caused transfer of calcein to cells farther from the donor cells **(** **Fig. 2**), suggesting a vibration induced increase in the transfer efficiency of gap junctions. We confirmed that the observed increase in GJIC was facilitated through gap junctions by blocking gap junctions for three hours with 18α-GA gap junction blocker. Application of 75 µM 18α-GA was not toxic to MLO-Y4 cells with 98.8% of the cell population remaining viable compared to DMSO treated controls (data not shown). Blocking gap junction function decreased calcein transference by approximately 80% (p<0.0001) (**Fig. 3**).

Although the number of GJIC+ cells was not significantly different between the vibrated groups, we correlated the results from GJIC experiments with mechanical variables from our FE model, including vibration induced fluid shear, acceleration magnitude, and estimated nuclear displacement. Acceleration induced nuclear displacement, but not acceleration magnitude or fluid shear, was significantly correlated with the observed GJIC differences between groups (ρ = 0.28, p = 0.016).

### Akt signaling

**Figure 4:**
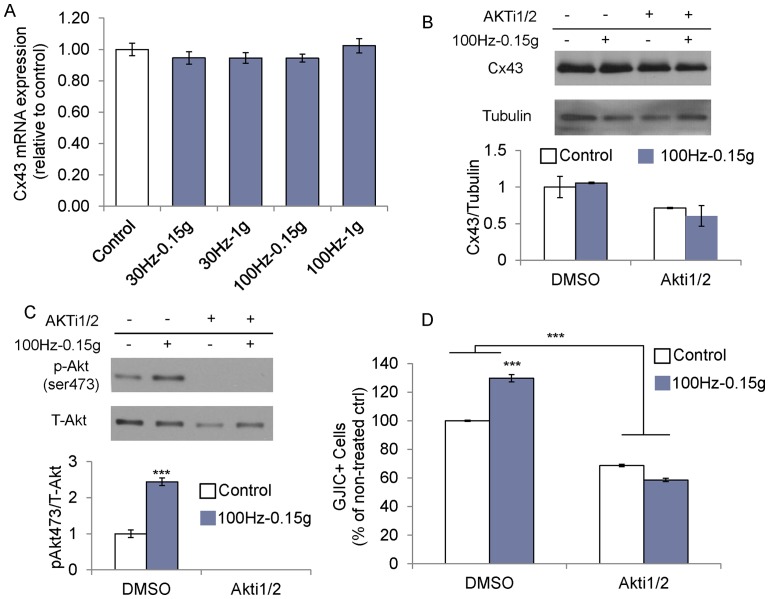
Vibration induced GJIC is controlled by Akt signaling. One hour after vibration treatment, (A) Cx43 mRNA levels remained unchanged. (B) Cx43 protein levels were also unaffected by vibrations (0.15 g–100 Hz) without and with Akt inhibition but (C) vibrations increased Akt phosphorylation (ser 473) 2.4-fold. (D) Inhibition of Akt activation also inhibited the vibration induced increase in calcein transference. ***: p<0.001 against control.

The increase in GJIC following vibration was not accompanied by an increase in Cx43 mRNA expression (**Fig. 4A**)**.** Fluid flow is known to increase Akt activation [33] but vibration induced fluid shear per se did not play a role in GJIC in this study. We therefore asked whether vibrations can increase Akt activation in the absence of significant fluid shear. To minimize fluid shear, we tested the vibration regime that produced the lowest levels of shear (100Hz-0.15 g, 0.04Pa). Cx43 protein levels remained unchanged after vibration exposure and were independent of Akt activation (**Fig. 4B**)**.** Akt phosphorylation (ser473) increased 2.4-fold (p<0.001) 1 h after vibration treatment (**Fig. 4C**), leading to a 29% (p<0.001) greater number of GJIC+ cells**.** 1 h pre-treatment of cells with Akt inhibitor AKTi1/2(40 µM) caused calcein transference to drop 31% below non-vibrated control levels (p<0.001). Further, oscillatory vibrations did not increase GJIC when Akt was inhibited (**Fig. 4D**).

## Discussion

We tested whether vibrations can increase osteocyte GJIC and if so, whether this increase is related to a specific mechanical variable altered by the application of oscillatory mechanical signals. During vibrations, cells were subject to both accelerations and fluid shear [43], [46]. We included vibration groups that created fluid shear up to 0.94Pa, a magnitude that is commonly used for fluid flow experiments [61], [62]. An FE model of an adherent cell established that accelerations per se led to much greater nuclear motions compared to vibration induced fluid shear which accounted for only 1% of total nuclear displacement. All vibration regimes caused significant increases in GJIC activity compared to non-vibrated controls. Fluid shear magnitude did not influence the outcome with no differences in calcein transference between the lowest (0.04Pa, 100 Hz−0.15 g) and highest fluid shear group (0.94Pa, 30 Hz−1 g). Thus, vibrations may aid in osteocyte gap junctional communication through nuclear motions induced directly by the transmitted oscillatory accelerations.

A dynamic FE model of an adherent cell was used to determine whether nuclear displacements may play a role in the mechanotransduction of vibrations. Even though the in silico data supported the hypothesis, simplifications and assumptions regarding the geometry and material properties of the cell need to be considered. Computational data have shown that the viscoelastic properties of the cytoplasm serve to dampen force transfer efficiency through the cytoskeleton by filtering certain frequencies [65]. Although we assumed that the high frequency of the mechanical signal justifies the use of elastic material properties, complex interactions between the viscous cytoplasm and the stiff cytoskeleton were not considered in our model. While outside the scope of our current study, we recognize that more realistic simulations that include a functional cytoskeleton will be critical towards accurately predicting cellular deformations under vibration. Additionally, the large difference in nuclear motions induced by fluid shear and acceleration could change significantly as a function of nucleus size, geometry, and density. This is less of a concern for this study as we focused on relative differences between fluid shear and accelerations but the true magnitude of nuclear displacement may turn out to be greatly different from data reported here. To address the concern of simplified cell properties, we tested a large range of cellular material properties. For instance, a range of 2.6–8.3kPa for the nuclear modulus of osteoblasts and osteocytes has been reported previously [58] and simulations of our model exceeded this range (3–18kPa). Together, we recognize the limitations of our idealized model but believe that it is important to provide preliminary data regarding the primary mode of cellular deformation during vibrations.

It is imperative to investigate the effects of vibratory mechanical signals in physiologically relevant models [21]. As a basic step towards this goal, we chose a simple 2D in vitro model that allows for the precise control of cellular fluid shear during the application of vibrations [46]. Even though the coupling of fluid shear with vibrations in our system is similar to the mechanical conditions of bone cells in vivo, a future study that completely separates vibratory effects from fluid shear will provide valuable data for the identification of specific differences in the cellular response to the two distinct mechanical signals. Factors including hormonal PTH and extracellular calcium concentration may alter Cx43 mediated GJIC [66], a process known to play an important role in bone formation and fracture healing in vivo [67], and inherently, our in vitro model cannot capture these factors. Further, the functions of osteocytes appear to be similar between different species [19] but it is clear that an in vitro model cannot replicate the 3D environment of an osteocytic network of any specie. Thus, conclusions from this 2D in vitro study cannot directly be extrapolated to ex-vivo and in vivo models. Finally, we did not monitor changes in Ca2+ or ATP levels in this study but mechanically induced GJIC in osteoblastic cells appears to be independent of Ca2+ signaling [10], [11]. In osteocytes (MLO-Y4 and in vivo), mechanical signals such as fluid flow and membrane stretch (hypotonic swelling) increase VSCC-dependent (voltage sensitive Ca2+ channels) ATP release mediated via ERK1/2 [12], [13]. Although outside the scope of current work, future studies that elucidate possible interactions between VSCC and Cx43 signaling will be important.

Unlike our previous studies with MSCs [48] and osteoblasts [46], GJIC in osteocytes was not modulated by the acceleration and frequency of the mechanical signal. We previously showed that increasing RhoA activity may increase the sensitivity of MSCs to vibrations and that transcriptional activity of cytoskeletal adaptor proteins is positively correlated with acceleration magnitude [48]. If mechanotransduction of vibrations comprises a response to forces generated within the cell, osteocytes appear to have a lower threshold for mechanosensing than MSCs as they responded to different accelerations and frequencies similarly. Although the reason for this differential cellular sensitivity is not clear, osteocytes have a more extensively developed cytoskeleton than MSCs and are therefore stiffer [53]. While our FE model suggested that greater cell stiffness decreases nuclear motions, a stiffer cytoskeleton can more effectively transmit forces [63], perhaps causing osteocytes to more readily sense mechanical signals than MSCs [64]. Thus, it is conceivable that all mechanical signals tested in this study exceeded an osteocytic response threshold, giving rise to the lack of differences between the vibration groups.

Nuclear motions within the cell impose forces on the cytoskeleton, ostensibly initiating mechanotransduction pathways including integrin related signaling. Akt signaling plays an important role in activating cellular sensing involving the cytoskeleton and formation of new focal adhesions and preserving cellular β-catenin levels in response to mechanical stretch [37], [68], [69]. Here, we showed that vibrations increase Akt activation in MLO-Y4 cells. Exposure to vibrations not only increased Akt activation but inhibiting Akt activation also inhibited the vibration induced increase in GJIC, suggesting that vibration induced phosphorylation of Akt modulates Cx43 function. In MLO-Y4 cells, fluid flow induced PI3K signaling increases Akt activation [33], potentially regulating Cx43 function through integrins [70] while in myocytes, β-catenin co-localizes with Cx43, increasing gap junction related Ca2+ wave propagation speed [71]. Perhaps, vibrations enhance Cx43 function as a downstream of Akt signaling through integrins or Cx43/β–catenin co-localization [71], a hypothesis to be tested in future investigations.

We showed that independent of the magnitude of generated fluid shear, vibrations can raise GJIC in osteocytes and that this increase is dependent on Akt signaling. These results indicate that at the cellular level, high-frequency acceleratory signals can not only activate cell signaling that may ultimately alter protein production but also contain basic information that enhances cellular communication. If these data can be extrapolated to in vivo models, our results imply that vibrations may modulate cell metabolism not just locally but by orchestrating a response through the through lacunar-canalicular network, they may elicit a response across larger regions. Highlighting the important role of GJIC in communicating anabolic signals, loss of Cx43 in osteocytes results in delayed fracture healing and bone formation [67]. Our mechanical cell model exposed to vibrations of different frequencies and magnitude was consistent with the hypothesis that nuclear motions but not fluid shear are involved in converting mechanical information into biochemical signals. Whether the anabolic [72] and anti-catabolic [42] effects of vibrations or the vibration induced increase in cellular sensitivity to mechanical [73] or biochemical signals [74] can be ascribed to more efficient signaling between osteocytes is yet to be determined.

## References

[1] Bergoffen J, Scherer S, Wang S, Scott M, Bone L, et al (1993) Connexin mutations in X-linked Charcot-Marie-Tooth disease. Science 262: 2039–2042. 8266101 10.1126/science.8266101

[2] Simon AM, Goodenough DA, Li E, Paul DL (1997) Female infertility in mice lacking connexin 37. Nature 385: 525–529. 9020357 10.1038/385525a0

[3] Kelsell DP, Dunlop J, Stevens HP, Lench NJ, Liang JN, et al (1997) Connexin 26 mutations in hereditary non-syndromic sensorineural deafness. Nature 387: 80–83. 9139825 10.1038/387080a0

[4] Ilvesaro J, Vaananen K, Tuukkanen J (2000) Bone-resorbing osteoclasts contain gap-junctional connexin-43. Journal of Bone and Mineral Research 15: 919–926. 10804022 10.1359/jbmr.2000.15.5.919

[5] Schirrmacher K, Schmitz I, Winterhager E, Traub O, Brummer F, et al (1992) Characterization of gap junctions between osteoblast-like cells in culture. Calcified Tissue International 51: 285–290. 1330238 10.1007/BF00334489

[6] Civitelli R, Beyer EC, Warlow PM, Robertson AJ, Geist ST, et al (1993) Connexin43 mediates direct intercellular communication in human osteoblastic cell networks. The Journal of Clinical Investigation 91: 1888–1896. 8387535 10.1172/JCI116406 PMC288182

[7] Jones SJ, Gray C, Sakamaki H, Arora M, Boyde A, et al (1993) The incidence and size of gap junctions between the bone cells in rat calvaria. Anatomy and Embryology 187: 343–352. 8390141 10.1007/BF00185892

[8] Kamioka H, Ishihara Y, Ris H, Murshid SA, Sugawara Y, et al (2007) Primary Cultures of Chick Osteocytes Retain Functional Gap Junctions between Osteocytes and between Osteocytes and Osteoblasts. Microscopy and Microanalysis 13: 108–117. 17367550 10.1017/S143192760707016X

[9] Nomura S, Takano-Yamamoto T (2000) Molecular events caused by mechanical stress in bone. Matrix Biology 19: 91–96. 10842092 10.1016/s0945-053x(00)00050-0

[10] Bacabac RG, Smit TH, Mullender MG, Dijcks SJ, Van Loon JJWA, et al (2004) Nitric oxide production by bone cells is fluid shear stress rate dependent. Biochemical and Biophysical Research Communications 315: 823–829. 14985086 10.1016/j.bbrc.2004.01.138

[11] Genetos DC, Kephart CJ, Zhang Y, Yellowley CE, Donahue HJ (2007) Oscillating fluid flow activation of gap junction hemichannels induces ATP release from MLO-Y4 osteocytes. Journal of Cellular Physiology 212: 207–214. 17301958 10.1002/jcp.21021 PMC2929812

[12] Cherian PP, Siller-Jackson AJ, Gu S, Wang X, Bonewald LF, et al (2005) Mechanical Strain Opens Connexin 43 Hemichannels in Osteocytes: A Novel Mechanism for the Release of Prostaglandin. Molecular Biology of the Cell 16: 3100–3106. 15843434 10.1091/mbc.E04-10-0912 PMC1165395

[13] Ajubi NE, Klein-Nulend J, Alblas MJ, Burger EH, Nijweide PJ (1999) Signal transduction pathways involved in fluid flow-induced PGE(2) production by cultured osteocytes. American Journal of Physiology-Endocrinology and Metabolism 276: E171–E178. 10.1152/ajpendo.1999.276.1.E171 9886964

[14] Li Z, Zhou Z, Yellowley CE, Donahue HJ (1999) Inhibiting gap junctional intercellular communication alters expression of differentiation markers in osteoblastic cells. Bone 25: 661–666. 10593410 10.1016/s8756-3282(99)00227-6

[15] Schiller PC, D'Ippolito G, Balkan W, Roos BA, Howard GA (2001) Gap-junctional communication is required for the maturation process of osteoblastic cells in culture. Bone 28: 362–369. 11336916 10.1016/s8756-3282(00)00458-0

[16] Lloyd SA, Loiselle AE, Zhang Y, Donahue HJ (2013) Shifting Paradigms on the Role of Connexin43 in the Skeletal Response to Mechanical Load. Journal of Bone and Mineral Research: n/a-n/a. 10.1002/jbmr.2165 PMC5949871 24588015

[17] Batra N, Kar R, Jiang JX (2012) Gap junctions and hemichannels in signal transmission, function and development of bone. Biochimica et Biophysica Acta (BBA) – Biomembranes 1818: 1909–1918. 21963408 10.1016/j.bbamem.2011.09.018 PMC3440861

[18] Jiang JX, Siller-Jackson AJ, Burra S (2007) Roles of gap junctions and hemichannels in bone cell functions and in signal transmission of mechanical stress. Frontiers in Bioscience 12: 1450–1462. 17127393 10.2741/2159 PMC1797155

[19] Sugawara Y, Ando R, Kamioka H, Ishihara Y, Honjo T, et al (2011) The Three-Dimensional Morphometry and Cell–Cell Communication of the Osteocyte Network in Chick and Mouse Embryonic Calvaria. Calcified Tissue International 88: 416–424. 21340572 10.1007/s00223-011-9471-7

[20] Saunders MM, You J, Trosko JE, Yamasaki H, Li Z, et al (2001) Gap junctions and fluid flow response in MC3T3-E1 cells. American Journal of Physiology – Cell Physiology 281: C1917–C1925. 11698250 10.1152/ajpcell.2001.281.6.C1917

[21] Chan M, Lu X, Huo B, Baik A, Chiang V, et al (2009) A Trabecular Bone Explant Model of Osteocyte–Osteoblast Co-Culture for Bone Mechanobiology. Cellular and Molecular Bioengineering 2: 405–415. 20827376 10.1007/s12195-009-0075-5 PMC2935082

[22] Grimston SK, Screen J, Haskell JH, Chung DJ, Brodt MD, et al.. (2006) Role of connexin43 in osteoblast response to physical load. In: Zaidi M, editor. Skeletal Development and Remodeling in Health, Disease, and Aging. Oxford: Blackwell Publishing. 214–224. 10.1196/annals.1346.023 16831921

[23] Alford AI, Jacobs CR, Donahue HJ (2003) Oscillating fluid flow regulates gap junction communication in osteocytic MLO-Y4 cells by an ERK1/2 MAP kinase-dependent mechanism. Bone 33: 64–70. 12919700 10.1016/s8756-3282(03)00167-4

[24] Turner CH, Robling AG, Duncan RL, Burr DB (2002) Do Bone Cells Behave Like a Neuronal Network? Calcified Tissue International 70: 435–442. 12149636 10.1007/s00223-001-1024-z

[25] Klein-Nulend J, Nijweide PJ, Burger EH (2003) Osteocyte and bone structure. Curr Osteoporos Rep 1: 5–10. 16036059 10.1007/s11914-003-0002-y

[26] Riddle RC, Donahue HJ (2009) From Streaming Potentials to Shear Stress: 25 Years of Bone Cell Mechanotransduction. Journal of Orthopaedic Research 27: 143–149. 18683882 10.1002/jor.20723

[27] Taylor AF, Saunders MM, Shingle DL, Cimbala JM, Zhou Z, et al (2007) Mechanically stimulated osteocytes regulate osteoblastic activity via gap junctions. American Journal of Physiology – Cell Physiology 292: C545–C552. 16885390 10.1152/ajpcell.00611.2005

[28] Yellowley CE, Li Z, Zhou Z, Jacobs CR, Donahue HJ (2000) Functional Gap Junctions Between Osteocytic and Osteoblastic Cells. Journal of Bone and Mineral Research 15: 209–217. 10703922 10.1359/jbmr.2000.15.2.209

[29] Batra N, Burra S, Siller-Jackson AJ, Gu SM, Xia XC, et al (2012) Mechanical stress-activated integrin alpha 5 beta 1 induces opening of connexin 43 hemichannels. Proceedings of the National Academy of Sciences of the United States of America 109: 3359–3364. 22331870 10.1073/pnas.1115967109 PMC3295295

[30] Cheng B, Zhao S, Luo J, Sprague E, Bonewald LF, et al (2001) Expression of Functional Gap Junctions and Regulation by Fluid Flow in Osteocyte-Like MLO-Y4 Cells. Journal of Bone and Mineral Research 16: 249–259. 11204425 10.1359/jbmr.2001.16.2.249

[31] Ziambaras K, Lecanda F, Steinberg TH, Civitelli R (1998) Cyclic Stretch Enhances Gap Junctional Communication Between Osteoblastic Cells. Journal of Bone and Mineral Research 13: 218–228. 9495514 10.1359/jbmr.1998.13.2.218

[32] Cherian PP, Cheng B, Gu S, Sprague E, Bonewald LF, et al (2003) Effects of Mechanical Strain on the Function of Gap Junctions in Osteocytes Are Mediated through the Prostaglandin EP2 Receptor. Journal of Biological Chemistry 278: 43146–43156. 12939279 10.1074/jbc.M302993200

[33] Xia XC, Batra N, Shi Q, Bonewald LF, Sprague E, et al (2010) Prostaglandin Promotion of Osteocyte Gap Junction Function through Transcriptional Regulation of Connexin 43 by Glycogen Synthase Kinase 3/beta-Catenin Signaling. Molecular and Cellular Biology 30: 206–219. 19841066 10.1128/MCB.01844-08 PMC2798309

[34] Watabe H, Furuhama T, Tani-Ishii N, Mikuni-Takagaki Y (2011) Mechanotransduction activates alpha(5)beta(1) integrin and PI3K/Akt signaling pathways in mandibular osteoblasts. Experimental Cell Research 317: 2642–2649. 21824471 10.1016/j.yexcr.2011.07.015

[35] Rangaswami H, Schwappacher R, Tran T, Chan GC, Zhuang S, et al (2012) Protein Kinase G and Focal Adhesion Kinase Converge on Src/Akt/β-Catenin Signaling Module in Osteoblast Mechanotransduction. Journal of Biological Chemistry 287: 21509–21519. 22563076 10.1074/jbc.M112.347245 PMC3375572

[36] Thompson WR, Guilluy C, Xie Z, Sen B, Brobst KE, et al (2013) Mechanically Activated Fyn Utilizes mTORC2 to Regulate RhoA and Adipogenesis in Mesenchymal Stem Cells. STEM CELLS 31: 2528–2537. 23836527 10.1002/stem.1476 PMC4040149

[37] Sen B, Xie Z, Case N, Thompson WR, Uzer G, et al.. (2013) mTORC2 regulates mechanically induced cytoskeletal reorganization and lineage selection in marrow derived mesenchymal stem cells. Journal of Bone and Mineral Research: n/a-n/a. 10.1002/jbmr.2031 PMC3870029 23821483

[38] Garman R, Gaudette G, Donahue LR, Rubin C, Judex S (2007) Low-level Accelerations applied in the absence of weight bearing can enhance trabecular bone formation. Journal of Orthopaedic Research 25: 732–740. 17318899 10.1002/jor.20354

[39] Judex S, Lei X, Han D, Rubin C (2007) Low-magnitude mechanical signals that stimulate bone formation in the ovariectomized rat are dependent on the applied frequency but not on the strain magnitude. Journal of Biomechanics 40: 1333–1339. 16814792 10.1016/j.jbiomech.2006.05.014

[40] Ozcivici E, Luu YK, Rubin CT, Judex S (2010) Low-level vibrations retain bone marrow's osteogenic potential and augment recovery of trabecular bone during reambulation. PLoS One 5: e11178. 20567514 10.1371/journal.pone.0011178 PMC2887365

[41] Lau E, Al-Dujaili S, Guenther A, Liu D, Wang L, et al (2010) Effect of low-magnitude, high-frequency vibration on osteocytes in the regulation of osteoclasts. Bone 46: 1508–1515. 20211285 10.1016/j.bone.2010.02.031 PMC3084034

[42] Sen B, Xie Z, Case N, Styner M, Rubin CT, et al (2011) Mechanical signal influence on mesenchymal stem cell fate is enhanced by incorporation of refractory periods into the loading regimen. Journal of Biomechanics 44: 593–599. 21130997 10.1016/j.jbiomech.2010.11.022 PMC3042527

[43] Coughlin TR, Niebur GL (2012) Fluid shear stress in trabecular bone marrow due to low-magnitude high-frequency vibration. Journal of Biomechanics 45: 2222–2229. 22784651 10.1016/j.jbiomech.2012.06.020

[44] Dickerson DA, Sander EA, Nauman EA (2008) Modeling the mechanical consequences of vibratory loading in the vertebral body: microscale effects. Biomechanics and Modeling in Mechanobiology 7: 191–202. 17520305 10.1007/s10237-007-0085-y

[45] Ozcivici E, Garman R, Judex S (2007) High-frequency oscillatory motions enhance the simulated mechanical properties of non-weight bearing trabecular bone. Journal of Biomechanics 40: 3404–3411. 17655852 10.1016/j.jbiomech.2007.05.015

[46] Uzer G, Manske S, Chan M, Chiang F-P, Rubin C, et al (2012) Separating Fluid Shear Stress from Acceleration during Vibrations In Vitro: Identification of Mechanical Signals Modulating the Cellular Response. Cellular and Molecular Bioengineering 5: 266–276. 23074384 10.1007/s12195-012-0231-1 PMC3466610

[47] Bacabac RG, Smit TH, Van Loon JJWA, Doulabi BZ, Helder M, et al (2006) Bone cell responses to high-frequency vibration stress: does the nucleus oscillate within the cytoplasm? FASEB J 20: 858–864. 16675843 10.1096/fj.05-4966.com

[48] Uzer G, Pongkitwitoon S, Ete Chan M, Judex S (2013) Vibration induced osteogenic commitment of mesenchymal stem cells is enhanced by cytoskeletal remodeling but not fluid shear. Journal of Biomechanics 46: 2296–2302. 23870506 10.1016/j.jbiomech.2013.06.008 PMC3777744

[49] Kato Y, Windle JJ, Koop BA, Mundy GR, Bonewald LF (1997) Establishment of an Osteocyte-like Cell Line, MLO-Y4. Journal of Bone and Mineral Research 12: 2014–2023. 9421234 10.1359/jbmr.1997.12.12.2014

[50] McGarry JG, Klein-Nulend J, Mullender MG, Prendergast PJ (2004) A comparison of strain and fluid shear stress in stimulating bone cell responses - a computational and experimental study. Faseb Journal 18: 482−+. 10.1096/fj.04-2210fje 15625080

[51] McGarry JG, Prendergast PJ (2004) A three-dimensional finite element model of an adherent eukaryotic cell. European cells & materials 7: 27–33 discussion 33–24. 15095253 10.22203/ecm.v007a03

[52] Verbruggen SW, Vaughan TJ, McNamara LM (2012) Strain amplification in bone mechanobiology: a computational investigation of the in vivo mechanics of osteocytes. Journal of the Royal Society Interface 9: 2735–2744. 10.1098/rsif.2012.0286 PMC3427527 22675160

[53] Darling EM, Topel M, Zauscher S, Vail TP, Guilak F (2008) Viscoelastic properties of human mesenchymally-derived stem cells and primary osteoblasts, chondrocytes, and adipocytes. Journal of Biomechanics 41: 454–464. 17825308 10.1016/j.jbiomech.2007.06.019 PMC2897251

[54] Dick DAT, Fry DJ, John PN, Rogers AW (1970) Autoradiographic demonstration of inhomogeneous distribution of sodium in single oocytes of Bufo bufo. Journal of Physiology-London 210: 305-&. 10.1113/jphysiol.1970.sp009212 PMC1395571 5501264

[55] Gouw TH, Vlugter JC (1966) Physical Properties of Triglycerides. I. Density and Refractive Index. Fette, Seifen, Anstrichmittel 68: 544–549.

[56] Heinrich V, Waugh RE (1996) A piconewton force transducer and its application to measurement of the bending stiffness of phospholipid membranes. Annals of Biomedical Engineering 24: 595–605. 8886240 10.1007/BF02684228

[57] Guilak F, Tedrow JR, Burgkart R (2000) Viscoelastic Properties of the Cell Nucleus. Biochemical and Biophysical Research Communications 269: 781–786. 10720492 10.1006/bbrc.2000.2360

[58] Sugawara Y, Ando R, Kamioka H, Ishihara Y, Murshid SA, et al (2008) The alteration of a mechanical property of bone cells during the process of changing from osteoblasts to osteocytes. Bone 43: 19–24. 18424248 10.1016/j.bone.2008.02.020

[59] Kato Y, Windle JJ, Koop BA, Mundy GR, Bonewald LF (1997) Establishment of an Osteocyte-like Cell Line, MLO-Y4. Journal of bone and mineral research 12: 2014–2023. 9421234 10.1359/jbmr.1997.12.12.2014

[60] Livak KJ, Schmittgen TD (2001) Analysis of Relative Gene Expression Data Using Real-Time Quantitative PCR and the 2-[Delta][Delta]CT Method. Methods 25: 402–408. 11846609 10.1006/meth.2001.1262

[61] You J, Yellowley CE, Donahue HJ, Zhang Y, Chen Q, et al (2000) Substrate deformation levels associated with routine physical activity are less stimulatory to bone cells relative to loading-induced oscillatory fluid flow. Journal of Biomechanical Engineering-Transactions of the Asme 122: 387–393. 10.1115/1.1287161 11036562

[62] Bancroft GN, Sikavitsast VI, van den Dolder J, Sheffield TL, Ambrose CG, et al (2002) Fluid flow increases mineralized matrix deposition in 3D perfusion culture of marrow stromal osteloblasts in a dose-dependent manner. Proceedings of the National Academy of Sciences of the United States of America 99: 12600–12605. 12242339 10.1073/pnas.202296599 PMC130506

[63] Hu SH, Chen JX, Butler JP, Wang N (2005) Prestress mediates force propagation into the nucleus. Biochemical and Biophysical Research Communications 329: 423–428. 15737604 10.1016/j.bbrc.2005.02.026

[64] Klein-Nulend J, van der Plas A, Semeins CM, Ajubi NE, Frangos JA, et al (1995) Sensitivity of osteocytes to biomechanical stress in vitro. FASEB J 9: 441–445. 7896017 10.1096/fasebj.9.5.7896017

[65] Shafrir Y, Forgacs G (2002) Mechanotransduction through the cytoskeleton. American Journal of Physiology-Cell Physiology 282: C479–C486. 11832332 10.1152/ajpcell.00394.2001

[66] Ishihara Y, Kamioka H, Honjo T, Ueda H, Takano-Yamamoto T, et al (2008) Hormonal, pH, and Calcium Regulation of Connexin 43–Mediated Dye Transfer in Osteocytes in Chick Calvaria. Journal of Bone and Mineral Research 23: 350–360. 17997713 10.1359/jbmr.071102

[67] Loiselle AE, Paul EM, Lewis GS, Donahue HJ (2013) Osteoblast and osteocyte-specific loss of Connexin43 results in delayed bone formation and healing during murine fracture healing. Journal of Orthopaedic Research 31: 147–154. 22718243 10.1002/jor.22178 PMC3640531

[68] Case N, Ma M, Sen B, Xie Z, Gross TS, et al (2008) β-Catenin Levels Influence Rapid Mechanical Responses in Osteoblasts. Journal of Biological Chemistry 283: 29196–29205. 18723514 10.1074/jbc.M801907200 PMC2570859

[69] Sen B, Guilluy C, Xie Z, Case N, Styner M, et al (2011) Mechanically induced focal adhesion assembly amplifies anti-adipogenic pathways in mesenchymal stem cells. Stem Cells 29: 1829–1836. 21898699 10.1002/stem.732 PMC3588570

[70] Batra N, Burra S, Siller-Jackson AJ, Gu S, Xia X, et al (2012) Mechanical stress-activated integrin α5β1 induces opening of connexin 43 hemichannels. Proceedings of the National Academy of Sciences 109: 3359–3364. 10.1073/pnas.1115967109 PMC3295295 22331870

[71] Ai Z, Fischer A, Spray DC, Brown AMC, Fishman GI (2000) Wnt-1 regulation of connexin43 in cardiac myocytes. The Journal of Clinical Investigation 105: 161–171. 10642594 10.1172/JCI7798 PMC377428

[72] Tirkkonen L, Halonen H, Hyttinen J, Kuokkanen H, Sievanen H, et al (2011) The effects of vibration loading on adipose stem cell number, viability and differentiation towards bone-forming cells. Journal of the Royal Society Interface 8: 1736–1747. 10.1098/rsif.2011.0211 PMC3203488 21613288

[73] Tanaka SM, Li J, Duncan RL, Yokota H, Burr DB, et al (2003) Effects of broad frequency vibration on cultured osteoblasts. Journal of Biomechanics 36: 73–80. 12485640 10.1016/s0021-9290(02)00245-2

[74] Patel MJ, Chang KH, Sykes MC, Talish R, Rubin C, et al (2009) Low Magnitude and High Frequency Mechanical Loading Prevents Decreased Bone Formation Responses of 2T3 Preosteoblasts. Journal of Cellular Biochemistry 106: 306–316. 19125415 10.1002/jcb.22007 PMC2737721

